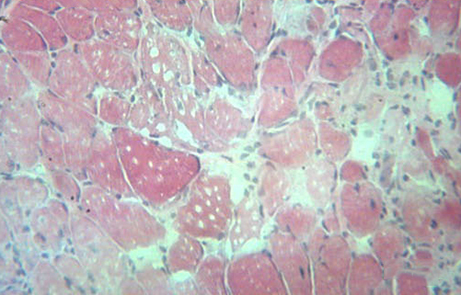# Promoting skeletal muscle regeneration in mouse and fly models of DMD

**Published:** 2014-01

Duchenne muscular dystrophy (DMD) causes progressive muscle weakness. There is currently no cure for the disease; however, recent studies have suggested that a bioactive lipid, sphingosine-1-phosphate (S1P), might protect against eventual muscle degeneration. S1P is an inhibitor of histone deacetylases (HDACs), which could form the molecular basis of its protective role in DMD. To test this possibility, Hannele Ruohola-Baker and colleagues exploited *Drosophila* and mouse models of the disease. They reduced levels of Rpd3, the *Drosophila* homologue of HDAC2, and found that disease symptoms were alleviated. In DMD mice, symptoms were also improved upon treatment with a compound that increases levels of S1P and suppresses HDAC activity. Interestingly, this improvement correlated with the expression of two miRNAs involved in promoting regeneration and energy metabolism in skeletal muscle tissue. This cross-species analysis thereby provides important insights into the pathology of DMD and the therapeutic potential of S1P. Page 41

**Figure:**